# Programmed cell death-1 blockade in kidney carcinoma may induce eosinophilic granulomatosis with polyangiitis: a case report

**DOI:** 10.1186/s12890-020-01375-5

**Published:** 2021-01-06

**Authors:** Masanori Harada, Hyogo Naoi, Kazuyo Yasuda, Yutaro Ito, Namio Kagoo, Tsutomu Kubota, Koshiro Ichijo, Eisuke Mochizuki, Masahiro Uehara, Shun Matsuura, Masaru Tsukui, Naoki Koshimizu

**Affiliations:** 1 Department of Respiratory Medicine, Fujieda Municipal General Hospital, 4-1-11 Surugadai, Fujieda City, Shizuoka Province Japan; 2 Department of Pathology, Fujieda Municipal General Hospital, 4-1-11 Surugadai, Fujieda City, Shizuoka Province Japan

**Keywords:** Programmed cell death-1, Programmed cell death ligand 1, Programmed cell death ligand 2, Airway hyper-reactivity, Nivolumab

## Abstract

**Background:**

Immune checkpoint inhibitors have potential applications in treating various cancers but are associated with immune-related adverse events, such as inflammation, in a wide range of organs; however, allergic inflammation caused by these agents has not been extensively studied.

**Case presentation:**

A 65-year-old man was diagnosed with a kidney neuroendocrine carcinoma. Three months after kidney resection surgery, the tumor cells had metastasized to his liver and lymph nodes. Subsequently, the patient started chemotherapy; however, regardless of treatment, the tumor grew, and the patient experienced a series of adverse effects, such as taste disorder, anorexia, and general fatigue. Finally, he was administered a programmed cell death (PD)-1 inhibitor, nivolumab (biweekly, toal 200 mg/body), which was effective against kidney carcinoma. However, the patient had a bronchial asthma attack at 22 cycles of nivolumab treatment and chest computed tomography (CT) revealed an abnormal bilateral shadow after 37 cycles of nivolumab treatment. Bronchoscopy findings revealed eosinophil infiltration in the lungs along with severe alveolar hemorrhage. Paranasal sinus CT scanning indicated sinusitis and nerve conduction analysis indicated a decrease in his right ulnar nerve conduction velocity. Based on these findings, the patient was diagnosed with eosinophilic granulomatosis with polyangiitis; he was treated with prednisolone, which alleviated his bronchial asthma. To restart nivolumab treatment, the dose of prednisolone was gradually tapered, and the patient was administered a monthly dose of mepolizumab and biweekly dose of nivolumab. To date, there have been no bronchial attacks or CT scan abnormalities upon follow up.

**Conclusions:**

We present a rare case in which a patient with cancer was diagnosed with eosinophilic granulomatosis with polyangiitis following treatment with a PD-1 inhibitor. Blockade of PD-1 and the programmed cell death ligand (PD-L) 1/PD-1 and PD-L2/PD-1 signaling cascade may cause allergic inflammation. Further studies are needed to identify the specific mechanisms underlying allergic inflammation after PD-1 blockade.

## Background

Immune checkpoint inhibitors (ICIs) have been employed to treat several cancers. The ICI nivolumab shows inhibitory effects by blocking immune system suppressors, such as programmed cell death protein 1 (PD-1), and decreasing T helper cell signaling [1]. However, nivolumab is also associated with several immune-related adverse events (irAEs) [2]. Among them, immune-related eosinophilia cases in patients treated with anti-PD-1 or anti-programmed cell death ligand (PD-L) 1 are rare, with an estimated frequency of only 2.9–3.3% [3, 4]. Eosinophil-induced adverse events (Eo-irAEs) occur in almost half of these cases. The most frequently damaged organ is the skin, followed by the lungs; eosinophilic pneumonia or bronchitis occurs in only 0.3% of cases. These allergic irAEs associated with ICIs have rarely been reported in the literature.

Here, we report a case of a patient with cancer who was treated with nivolumab and subsequently developed eosinophilic granulomatosis with polyangiitis (EGPA).

## Case presentation

A 65-year-old Japanese man was diagnosed with kidney large cell neuroendocrine carcinoma (G3 grade, 52.1% Ki67-positive stained cells, PD-L1 TPS 70%), and had undergone total left kidney resection and descending colectomy 3 years prior to visiting our hospital. Three months after surgery, the tumor cells had metastasized to his liver and lymph nodes around the abdominal aorta. At the time, there was no established treatment against neuroendocrine cell carcinoma of the renal cells in Japan. The patient provided written informed consent and started chemotherapy. First, he was administered two courses of carboplatin plus irinotecan; however, the tumor size increased. The patient then started second-line treatment with sunitinib, which was discontinued after 3 months because he developed a taste disorder and watery diarrhea. Next, the patient was administered everolimus as a third-line therapy; however, the patient developed anorexia and general fatigue, and treatment with everolimus was discontinued. Because there were no other standard therapies for treating neuroendocrine kidney carcinoma, bi-weekly nivolumab (200 mg/body) treatment was administered to the patient. After nivolumab treatment, the tumor gradually disappeared, and no adverse events other than a mildly increased peripheral absolute eosinophil count (300–500/μL) were observed after 5 cycles of nivolumab treatment.

**Fig. 1:**
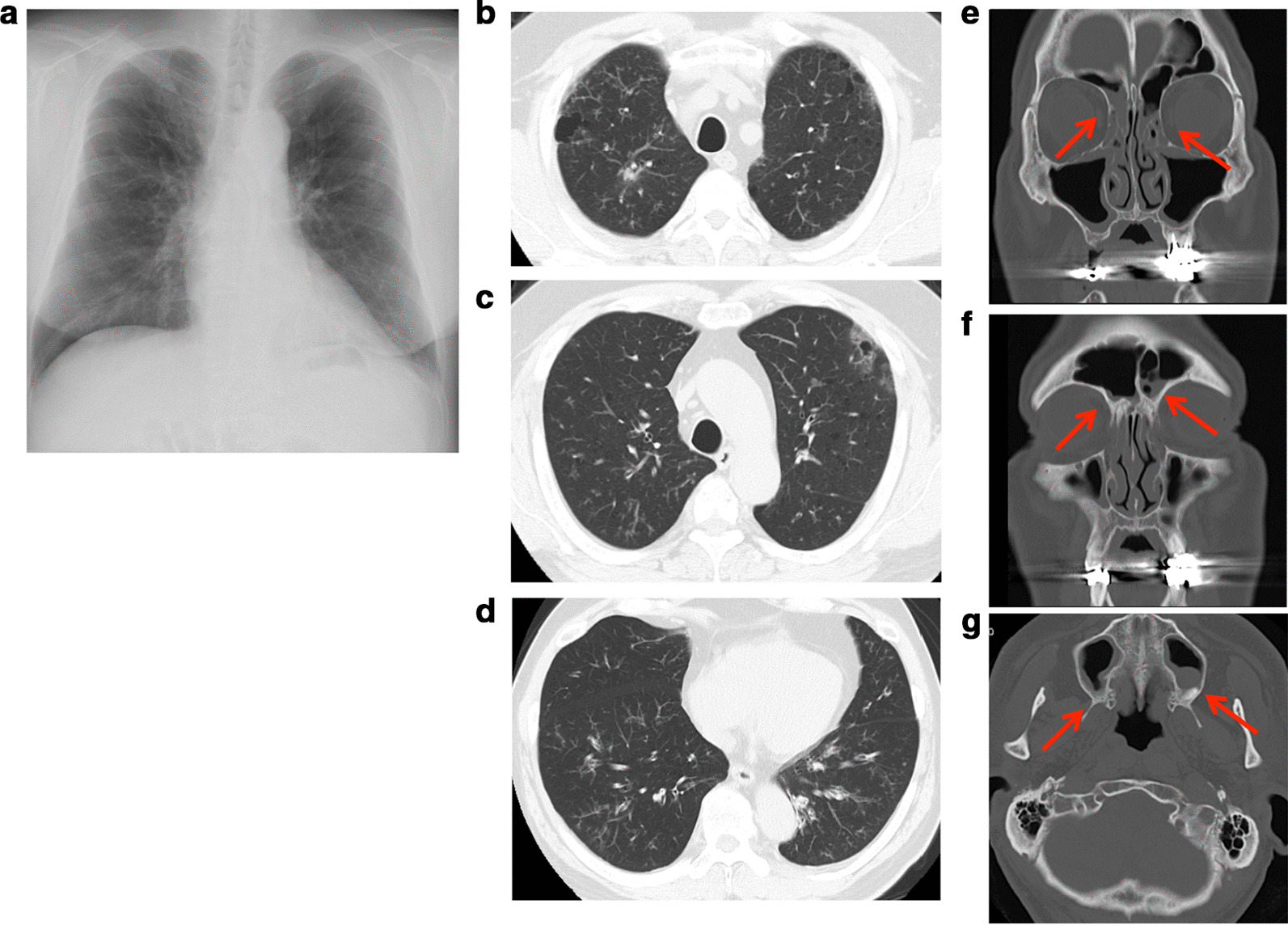
Thoracic radiogram and computed tomography (CT) scan at the bronchoscopy exam. Chest radiogram (**a**) showed bilateral linear shadow. Thoracic CT scan of upper (**b**, **c**) and lower lobes (**d**) showed bilateral ground-glass opacity. **e**–**g** Paranasal sinus CT scan; Ethmoid sinus (**e**), frontal sinus (**f**) and maxillary sinus (**g**). Each arrow indicates nasal mucosa thickening and fluid collection in the sinus. These appearances suggest paranasal sinusitis

**Fig. 2:**
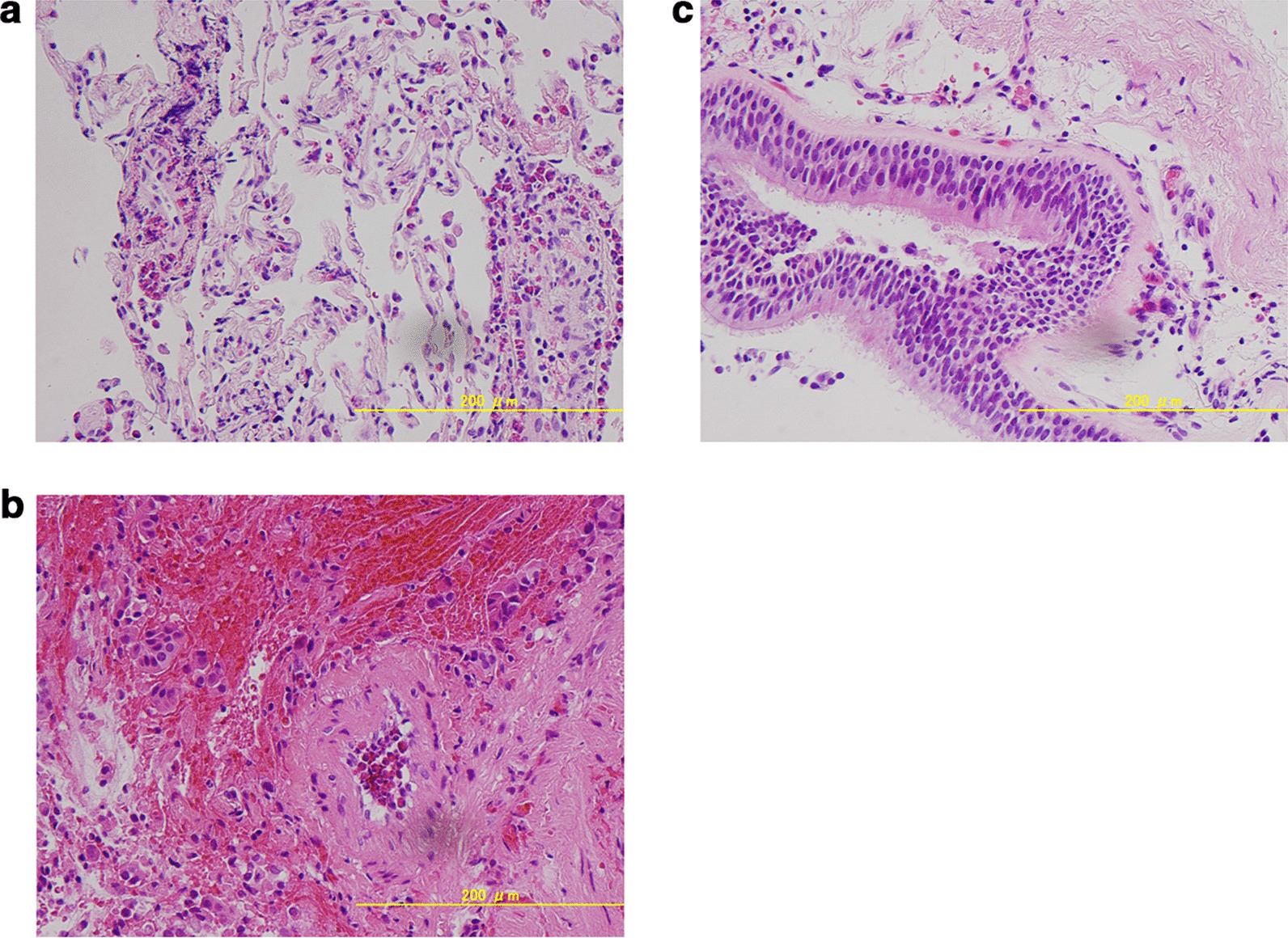
**a**, **b** Transbronchial lung biopsy sample from right lower lung (right B^8^); sample stained with hematoxylin and eosin (HE) and viewed at high power. **c** Airway mucosal biopsy sample from secondary carina; sample stained with HE and viewed at high power. Bar indicates 200 μm. **a** shows small blood vessel with neutrophil and eosinophil infiltration. Alveolar septa are thickened. **b** Shows lots of red blood cells on the lung tissue and intra-alveolar bleeding with septal edema. **c** Shows smooth muscle hyperplasia and thickened basement membrane with eosinophil infiltration

**Fig. 3:**
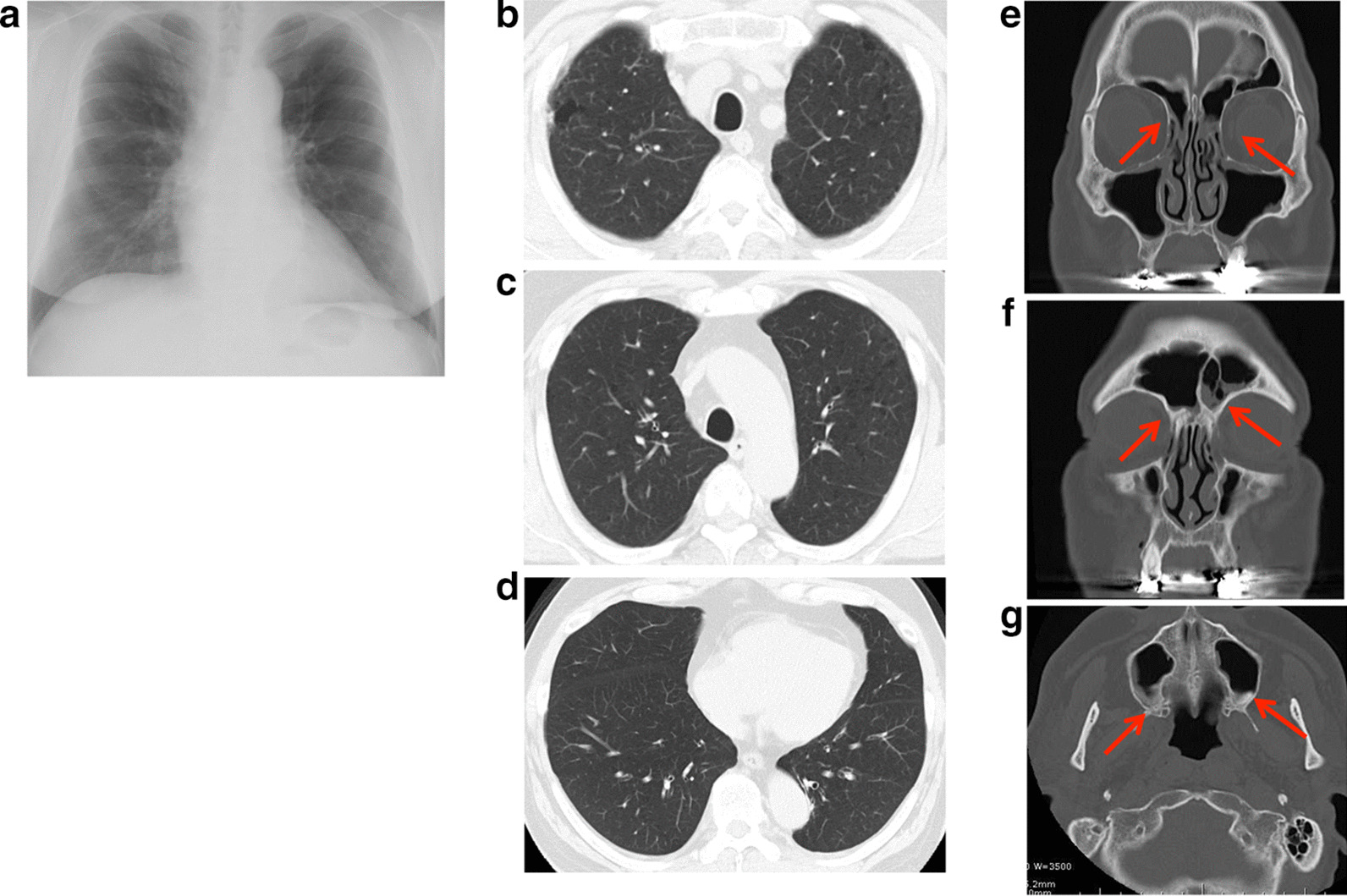
Thoracic radiogram (**a**), CT scans of chest (**b**–**d**) and ethmoid sinus (**e**), frontal sinus (**f**) and maxillary sinus (**g**) after a year from initiation of treatment for EGPA. Chest radiogram (**a**) shows no any abnormal shadows. Thoracic CT scan of upper (**b**, **c**) and lower lobes (**d**) also does not show any ground-glass opacity. Each arrow in **e**–**g** indicates nasal mucosa thickening and fluid collection that was shown in **e**–**g**

The patient experience his first bronchial asthma attack at 22 cycles of nivolumab treatment and was treated with a short course of corticosteroid burst therapy with 20 mg prednisolone along with inhalation therapy with budesonide/formoterol fumarate. This bronchial asthma attack was thought to be an adverse event associated with nivolumab; however, the patient continued the same dose of nivolumab treatment until 37 cycles because a good response against the kidney neuroendocrine carcinoma had been achieved. This clinical course is summarized in the Additional file 1: supplementary figure. Although nivolumab treatment had been discontinued until 37 cycles, the absolute eosinophil count remained high (26.6%, 1782.2 cells/μL) and his serum immunoglobulin E (IgE) levels were increased (924 IU/mL). Titers of anti-nuclear antibodies and anti-neutrophil cytoplasmic antibodies remained in the normal range. An IgE-radioallergosorbent test detected normal IgE levels, except against Japanese cedar and white cedar pollen. His fractional exhaled nitric oxide level was elevated to 107 ppb (normal range: 15–37 ppb). The patient had no history of allergy: however, all of his children had allergic bronchial asthma. He also showed dyspnea on exertion, developed sputum, and exhibited swelling and pain in his bilateral fingers. There were no other physical findings, including wheezing. Chest X-ray and computed tomography (CT) scanning detected a bilateral linear shadow which was not detected at his first visit to our hospital (Fig. 1a–d), and paranasal sinus CT scanning showed inflamed ethmoid, frontal, and maximal sinuses, indicating sinusitis (Fig. 1e–g). Although he had a normal respiratory function before initiating nivolumab treatment (FEV_1.0_ = 3.08 L, FEV_1.0_/FVC = 71.0%), his respiratory function test indicated an obstructive ventilation disorder (FEV_1.0_ = 1.63 L, FEV_1.0_/FVC = 50.6%). These results suggested that he had either eosinophilic pneumonia or EGPA. To confirm our hypothesis, we performed a bronchoscopy; the bronchoalveolar lavage fluid collected from the right B^5^ contained 40% lymphocytes and 3% eosinophils. Histopathological examination of a transbronchial lung biopsy sample from the right B^8^ showed small blood vessel hyperplasia with neutrophil infiltration and thickening of the alveolar septa with prominent eosinophil infiltration. Large numbers of red blood cells were observed on the lung tissue along with intra-alveolar bleeding (Fig. 2a, b), suggesting distinct alveolar hemorrhage with eosinophilic pneumonia. However hemosiderin-laden macrophages were not detected by Berlin blue staining, suggesting that the alveolar hemorrhage was an occasional event. Because he had experienced several bronchial asthma attacks and was already being treated with medication, the representative clinical findings needed for the bronchial asthma diagnosis were likely masked. To confirm the pathological findings of bronchial asthma, transbronchial mucosal biopsy was conducted. A bronchial mucosal lesion of the secondary carina also showed smooth muscle hyperplasia and thickening of the basement membrane with eosinophil infiltration (Fig. 2c). These findings suggested that the patient had bronchial asthma, eosinophilic pneumonia, and small vessel vasculitis. Finally, nerve conduction analysis showed that his right ulnar nerve conduction velocity was slightly decreased. There were no brain metastatic diseases, cervical spondylosis, or abnormalities related to rheumatoid arthritis. Although we could not definitively diagnose his neurological abnormalities, EGPA was thought to be one cause of the neuropathy. According to these findings with small vessel vasculitis, the patient was diagnosed with EGPA as per the American College of Rheumatology criteria for EGPA [5]. Distinct alveolar hemorrhage was not severe in the clinical setting and no heart involvement was detected; thus, treatment with 20 mg/day oral prednisolone was started. After 3 months, his bronchial asthma was improved, and bilateral infiltration had disappeared, as shown in Fig. 3. Steroid therapy was gradually tapered; during its discontinuation to less than 10 mg/day, a biweekly dose of mepolizumab (300 mg/day) was started to treat the EGPA. Currently, the patient is being treated with both nivolumab and mepolizumab, his peripheral eosinophilia has almost disappeared, and his respiratory function has improved (FEV_1.0_ = 2.94 L, FEV_1.0_/FVC = 71.2%), with paranasal sinus CT scanning showed normal findings (Fig. 3) after a year of treatment. He appears to be stable except for his neuropathy; to date, there have been no bronchial asthma attacks or CT scan abnormalities (Fig. 3).

## Discussion and conclusions

We report a rare case in which a patient with kidney neuroendocrine carcinoma was diagnosed with EGPA after treatment with nivolumab (a PD-1 checkpoint inhibitor). ICIs, such as nivolumab, are used to treat several cancers and have been associated with immune-related eosinophilia. The exact mechanisms mediating Eo-irAEs are not fully understood but a few clinical cases related to Eo-irAEs have been reported [6–8] (Table 1). Eo-irAEs can occur in systemic organs after ICI therapy and damage various organs, including the skin, lung, kidney, heart, and biliary ducts [4]. In the cases shown in Table 1, the Eo-irAEs were limited to the respiratory tract and showed a duration of development of 2–12 months. In the current case, the initial Eo-irAE was bronchial asthma, which occurred 9 months after starting nivolumab treatment; however, eosinophil infiltration was systemic and damaged the lungs, paranasal sinus, and peripheral nervous system. Before treatment with nivolumab, the patient’s absolute eosinophil count was normal; therefore, nivolumab was thought to be the main cause of the Eo-irAE. In a recent case, ipilimumab and nivolumab therapy (which was continued after Eo-ir) led to systemic infiltration of peripheral eosinophils [9]; however, there was a lack of sufficient pathological evidence of lung eosinophil infiltration. In our case, pathological evidence revealed eosinophil infiltration around the small vessels in the lung tissues and severe alveolar hemorrhage. These findings indicated eosinophilic vasculitis and eosinophilic pneumonia and were confirmed by CT scanning, although the damage to the paranasal sinus and peripheral nervous system in the pathological findings may differ from the damage to the lungs.

**Table 1:** Clinical appearances of previous case reports and our case

Case reports	Johdai et al. 2018 [4]	Maeno et al. 2017 [5]	Donato et al. 2019 [3]	This case
Age/sex	62/M	50/M	47/M	67/M
Diagnosis	AEP	BA	ABPA	EGPA
BA histoly	−	−	+	−
Length to AE	2 M	6 M	12 M	9 M
Tumor	Lung-sq	Lung-adeno	Lung-adeno	Kidney-NEC
PD-L1	N.F	N.F	50%	70%
ICI	Nivo	Nivo	Pembro	Nivo
Treatment	PSL	PSL	PSL/VRCZ	PSL/mepolizumab
Efficacy	imp	imp	imp	imp

AEP: acute eosinophilic pneumonia, BA: bronchial asthma, ABPA: allergic bronchopulmonary aspergillaosis, EGPA: Eosinophlic Granulomatosis with Polyangitis, lung-sq: lung squamous cell carcinoma, lung-adeno: lung adenocarcinoma, kidney-NEC: kidney neuroendocrine carcinoma, N.F: no findings, Nivo: nivolumab, Pembro: pembrolizumab, PSL: prednisolone, VRCZ: voriconazol, imp: improvement

Prolonged nivolumab treatment induced not only bronchial asthma but also systemic eosinophilic infiltration in our patient. The Eo-irAEs were thought to be an allergic reaction due to PD-1 inhibition. The underlying pathological mechanisms remain unclear; however, recent evidence suggests several hypotheses such as PD-L2/PD-1 signaling inhibition or PD-L2 intrinsic dysfunction.

PD-1 is a negative regulator of CD4-positive T-cells. The ligands for PD-1 are PD-L1 (B7-H1) and PD-L2 (B7-DC), both of which belong to the B7-CD28 family [10]. PD-L1 is expressed by different resting cells, such as dendritic cells, macrophages, T-cells, and B-cells, whereas PD-L2 expression appears to be restricted to activated dendritic cells and macrophages [11]. Furthermore, only 40% homology has been reported among the two ligands [12]. Studies of the roles of PD-1 and its ligands PD-L1 and PD-L2 have yielded conflicting results in allergen-induced inflammation and airway hyper-reactivity (AHR). AHR is one of the characteristics of bronchial asthma; PD-L1 and PD-L2 show opposing functions in airway modulation. PD-L1/PD-1 induces AHR, whereas PD-L2/PD-1 blocks the initiation and progression of airway inflammation [13]. In our case, PD-1 blockade induced peripheral eosinophilia and bronchial asthma attacks. Therefore, the interaction of PD-L2 with PD-1 may be stronger than that of PD-L1.

Matsumoto et al. reported that PD-L2 blockade induced not only AHR and eosinophilia, but also increased the production of interleukin (IL)-5 and IL-13 and decreased the production of interferon-γ [14]. Oflazoglu et al. reported that exogenous PD-L2 administration in an in vivo mouse asthma model resulted in elevation of serum IgE levels, eosinophilic and lymphocytic infiltration, and production of IL-5 and IL-13 [15]. In our case, PD-1 blockade by nivolumab induced an increase in the production of both interferon-γ and Th2-type immune cytokines. In addition to PD-L2 intrinsic regulation, allergen-driven enhancement of PD-L2 also limits the secretion of IL-12 and leads to exacerbation of AHR via a PD-1 independent mechanism [16]. These results suggest unknown signaling cascades (other than PD-1 signaling) underlying the AHR mechanisms.

Finally, in our case, mepolizumab (an IL-5 inhibitor) was useful during nivolumab discontinuation. Because the IL-5 signaling cascade is thought to be an important factor related to Eo-irAEs [6], the use of mepolizumab may have prevented nivolumab from inducing Eo-irAEs in the patient. Therefore, it is necessary to investigate the effects of Eo-irAEs on systemic organs.

In summary, we reported a rare case in which nivolumab treatment for kidney neuroendocrine carcinoma induced recurrent bronchial asthma attacks and led to systemic eosinophilic inflammation and EGPA. Although the exact mechanisms underlying allergic inflammation after PD-1 blockade remain unclear, PD-L1/PD-1 and PD-L2/PD-1 signaling may be key in deciphering the mechanisms mediating allergic inflammation.

## Supplementary information

**Additional file 1.** A clinical coarse after initiation of nivolumab treatment. Treatment cycles of nivolumab shows on the upper black bar. Black triangle represents oral 30mg/day prednisolone treatments for bronchial asthma attacks. BA-AE: bronchial asthma attack, BUD/FP: budesonide/formoterol fumarate, Tio: Tiotropium bromide, solid line represents peripheral absolute eosinophil count (EOS). Tumor size of live metastasis was represented as maximum diameter in the bottom rectangle area.

## Data Availability

Not applicable.

## Abbreviations

CT: Computed tomography
EGPA: Eosinophilic granulomatosis with polyangiitis
Eo-ir: Immune-related eosinophilia
Eo-irAE: Eosinophil-induced adverse event
ICI: Immune checkpoint inhibitor
IL: Interleukin
IgE: Immunoglobulin E
irAE: Immune-related adverse event
PD-1: Programmed cell death 1
PD-L1: Programmed cell death ligand 1
PD-L2: Programmed cell death ligand 2
