# Supplementary material for: Programmed cell death-1 blockade in kidney carcinoma may induce eosinophilic granulomatosis with polyangiitis: a case report
Source: BMC Pulm Med. 2021 Jan 6;21:6. doi: 10.1186/s12890-020-01375-5 (PMC7789237; doi:10.1186/s12890-020-01375-5)
Supplement: Supplementary file 1 — Additional file 1. A clinical coarse after initiation of nivolumab treatment. Treatment cycles of nivolumab shows on the upper black bar. Black triangle represents oral 30mg/day prednisolone treatments for bronchial asthma attacks. BA-AE: bronchial asthma attack, BUD/FP: budesonide/formoterol fumarate, Tio: Tiotropium bromide, solid line represents peripheral absolute eosinophil count (EOS). Tumor size of live metastasis was represented as maximum diameter in the bottom rectangle area. [file 12890_2020_1375_MOESM1_ESM.pdf]

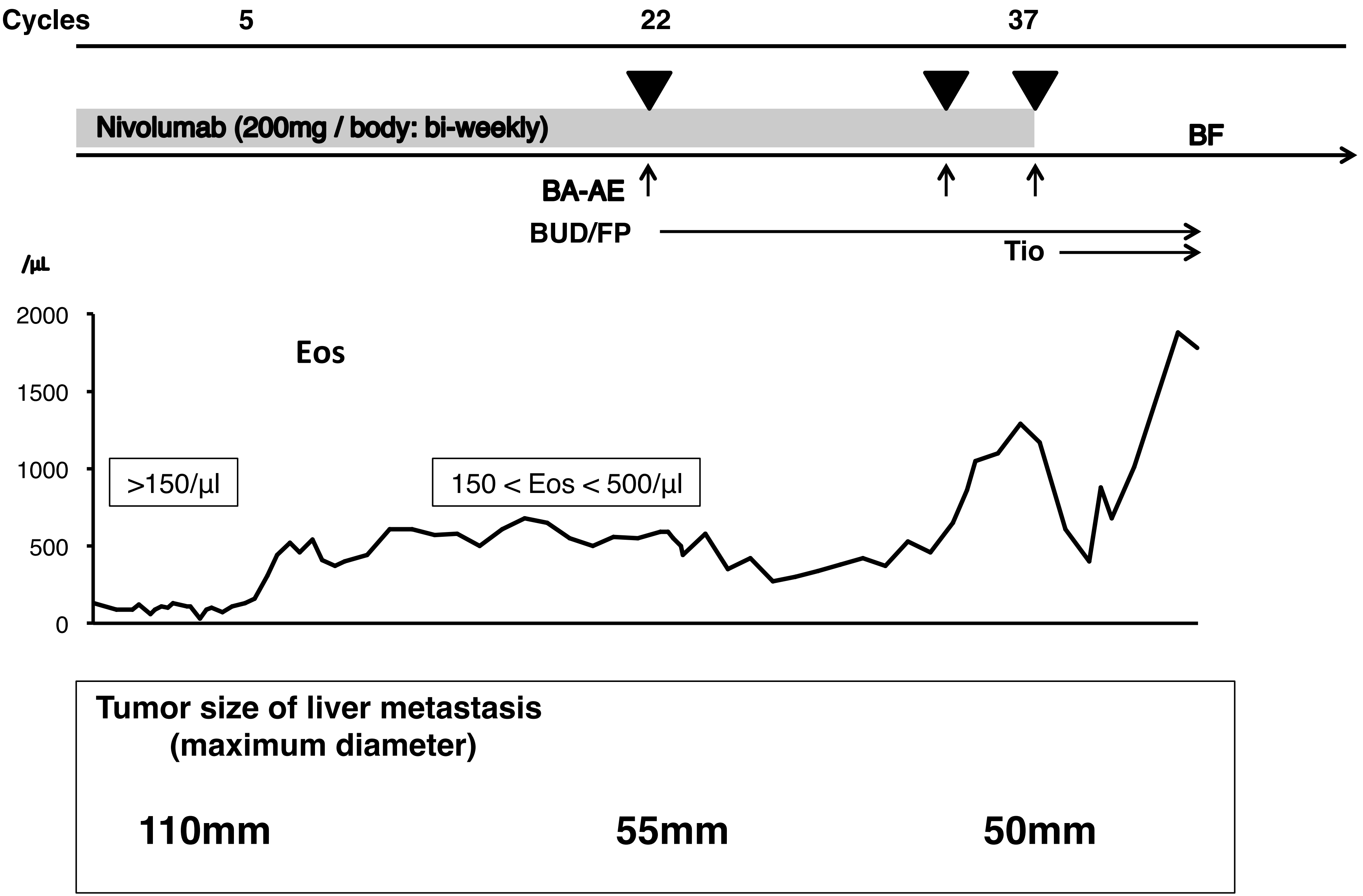
Cycles
5
22
37
Nivolumab (200mg / body: bi-weekly)
BF
BA-AE
BUD/FP
Tio
/μL
2000
1500
1000
500
0
Eos
>150/μl
150 < Eos < 500/μl
Tumor size of liver metastasis
(maximum diameter)
110mm
55mm
50mm
